# Inflammation in Target Organs: From the Bench to the Clinic

**DOI:** 10.3390/cells15080681

**Published:** 2026-04-13

**Authors:** Roni Weinberg Sibony, Itamar Raz

**Affiliations:** 1 Helen Schneider Hospital for Women, Rabin Medical Center-Beilinson Hospital, Petach Tikva 49100, Israel; 2 Faculty of Medicine, Hadassah Hebrew University Hospital, Jerusalem 91120, Israel

Metabolic disorders such as obesity, diabetes, dyslipidemia, and hypertension are increasingly recognized as systemic diseases that extend their pathological impact far beyond energy imbalance. These conditions drive inflammatory cascades that compromise the integrity and function of multiple target organs, including the heart, liver, kidneys, vascular system and brain. The intricate interplay between metabolism, oxidative stress, and inflammation constitutes a unifying mechanism of tissue injury and fibrotic transformation [1]. This Special Issue compiles recent advances that illuminate how inflammatory signaling networks orchestrate cellular damage across organs, providing mechanistic insights and identifying emerging therapeutic targets.

The collected studies delineate inflammation as a pivotal axis linking metabolic derangements to organ dysfunction. Standl and Schnell describe the expanding concept of a “cardio–renal–metabolic–cancer syndrome,” underscoring that hyperinsulinemia, insulin resistance, and low-grade inflammation not only promote diabetes and cardiovascular disease but also heighten the risk of malignancy. Their analysis emphasizes how ectopic fat accumulation, mitochondrial stress, and dysregulated insulin/IGF signaling establish a pro-inflammatory and pro-oncogenic environment, suggesting that metabolic correction may mitigate cancer risk alongside cardiometabolic benefit [2]. Complementing this systemic perspective, Pellegrini and colleagues map the “inflammatory trajectory” of type 2 diabetes, demonstrating that low-grade inflammation precedes hyperglycemia and persists despite glycemic control. They highlight how senescence, trained immunity, and residual inflammatory risk perpetuate vascular injury, while novel agents such as GLP-1 receptor agonists, SGLT2 inhibitors, and colchicine show potential in curbing this inflammatory continuum [3,4]. Furthermore, the deep pathogenic linkage within cardio–renal–metabolic–cancer syndrome warrants a proactive clinical shift toward screening for cancer immediately upon the diagnosis of diabetes, leveraging a critical window of opportunity to mitigate the heightened risk of malignancy in these patients.

At the vascular level, Cheng and Wang review inflammatory pathways in coronary artery disease, identifying NLRP3 inflammasome activation, IL-1 and IL-6 signaling, and complement cascades as key mediators of plaque instability. They analyze why certain anti-inflammatory strategies, such as low-dose colchicine or IL-1β inhibition, successfully reduce recurrent events whereas others fail, emphasizing the need for pathway-specific interventions [5]. In the realm of cardiac inflammation, Sur and co-workers provide experimental evidence that cytotoxic CD4^+^ T cells can directly mediate autoimmune myocarditis through IFN-γ- and granzyme-B-dependent mechanisms, revealing a novel effector subset with potential relevance to inflammatory cardiomyopathies [6,7].

Liver-centered investigations in this issue elucidate the convergence of metabolic stress and innate immunity. Babuta et al. demonstrate that combined metabolic dysfunction and alcohol exposure synergistically activate the NLRP3–IL-1β axis, promoting neutrophil infiltration and neutrophil extracellular trap (NET) formation [8]. Pharmacologic inhibition of NLRP3 with MCC950 markedly attenuated liver injury, identifying inflammasome signaling as a therapeutic entry point for metabolic alcohol-associated steatohepatitis [9]. Amer and colleagues further reveal that in patients with MASH, natural killer (NK) cell dysfunction correlates with suppressed STING pathway activity in advanced fibrosis. Restoration of STING signaling—particularly when combined with insulin receptor activation—reinvigorated NK cytotoxicity against hepatic stellate cells, suggesting an immunometabolic interface that governs fibrogenesis. Significantly, the integration of insulin signaling with STING activation exhibits a synergistic potential to reinvigorate NK cell cytotoxic functions, offering a powerful immunometabolic approach to reverse immune evasion and counteract fibrogenesis in advanced liver fibrosis [10].

The kidney represents another critical target of metabolic inflammation. Wojtacha and co-authors report that spontaneously hypertensive rats exhibit an age-dependent shift from suppressed to overactive renal inflammation, oxidative stress, and metabolic dysregulation, implicating the kidney as both a victim and amplifier of hypertensive injury [11,12]. In a complementary analysis of systemic shock physiology, Vázquez-Galán et al. integrate ischemia–reperfusion dynamics, oxidative stress, and immune imbalance, depicting the transition from compensatory to irreversible organ failure. Their conceptual framework underscores how immune dysregulation and redox imbalance cooperate to drive multi-organ dysfunction [13,14].

The heart, a recurring focal point, is revisited in the review by Amara, Stoler, and Birati, who synthesize evidence linking innate and adaptive immunity to heart failure pathogenesis. They delineate roles for cytokines, endothelial inflammation, and neurohormonal cross-talk in myocardial remodeling, while noting that clinical trials targeting single cytokines have yielded mixed outcomes, highlighting the complexity of inflammatory signaling within cardiac tissue [15].

Together, these contributions advance a holistic view of inflammation as the common denominator of metabolic organ damage. They reveal that inflammatory responses are not merely secondary to metabolic stress but integral to disease initiation, progression, and cross-organ communication. By dissecting molecular mediators—from NLRP3, IL-1β, and IL-6 to STING and HIF-1α—this collection highlights the interplay between immune pathways, mitochondrial dysfunction, and metabolic signaling. Importantly, several studies point to translational opportunities: selective inflammasome blockade, immune-metabolic modulation, senolytic and antioxidant therapies, and precise anti-cytokine interventions.

In summary, this Special Issue emphasizes the importance of conceptualizing metabolic disorders within an immuno-inflammatory framework. The integration of basic, translational, and clinical research presented herein provides a unified understanding of how chronic low-grade inflammation links metabolic dysregulation to fibrosis and organ failure. Elucidation of these shared mechanisms may facilitate the development of innovative therapeutic strategies aimed at interrupting inflammatory circuits at their metabolic origin, thereby transforming preventive and therapeutic approaches across cardio-renal-hepatic and systemic diseases.
